# Nitrogen Removal from Landfill Leachate by Microalgae

**DOI:** 10.3390/ijms17111926

**Published:** 2016-11-17

**Authors:** Sérgio F. L. Pereira, Ana L. Gonçalves, Francisca C. Moreira, Tânia F. C. V. Silva, Vítor J. P. Vilar, José C. M. Pires

**Affiliations:** 1 Laboratório de Engenharia de Processos, Ambiente e Energia (LEPABE), Departamento de Engenharia Química, Faculdade de Engenharia, Universidade do Porto, 4200-465 Porto, Portugal; ega11026@fe.up.pt (S.F.L.P.); pdeqb0707727@fe.up.pt (A.L.G.); 2 Laboratory of Separation and Reaction Engineering—Laboratory of Catalysis and Materials (LSRE-LCM), Departamento de Engenharia Química, Faculdade de Engenharia, Universidade do Porto, 4200-465 Porto, Portugal; francisca.moreira@fe.up.pt (F.C.M.); tania.silva@fe.up.pt (T.F.C.V.S.); vilar@fe.up.pt (V.J.P.V.)

**Keywords:** biomass production, *Chlorella vulgaris*, landfill leachate, microalgae, nutrient removal kinetics, wastewater treatment

## Abstract

Landfill leachates result from the degradation of solid residues in sanitary landfills, thus presenting a high variability in terms of composition. Normally, these effluents are characterized by high ammoniacal-nitrogen (N–NH_4_^+^) concentrations, high chemical oxygen demands and low phosphorus concentrations. The development of effective treatment strategies becomes difficult, posing a serious problem to the environment. Phycoremediation appears to be a suitable alternative for the treatment of landfill leachates. In this study, the potential of *Chlorella vulgaris* for biomass production and nutrients (mainly nitrogen and phosphorus) removal from different compositions of a landfill leachate was evaluated. Since microalgae also require phosphorus for their growth, different loads of this nutrient were evaluated, giving the following N:P ratios: 12:1, 23:1 and 35:1. The results have shown that *C. vulgaris* was able to grow in the different leachate compositions assessed. However, microalgal growth was higher in the cultures presenting the lowest N–NH_4_^+^ concentration. In terms of nutrients uptake, an effective removal of N–NH_4_^+^ and phosphorus was observed in all the experiments, especially in those supplied with phosphorus. Nevertheless, N–NO_3_^−^ removal was considered almost negligible. These promising results constitute important findings in the development of a bioremediation technology for the treatment of landfill leachates.

## 1. Introduction

At the present time, the disposal of solid waste material in sanitary landfills continues to be an extensively used option, since it still has short-term economic advantages when compared to other waste management alternatives [1]. However, during its long-term process of transforming waste into stabilized material, considerable volumes of gaseous and liquid effluents are produced and those constitute new problems to deal with.

Landfill leachate is a highly contaminated liquid that outcomes from a group of processes occurring within landfill cells: rainwater percolation, moisture accumulation and biochemical degradation [1,2]. Its ability to induce lethal and pre-pathological alterations on human cells [3,4], mice [5], marine organisms [6,7] and plants [8] is well known and, as a consequence, its proper treatment is imperative. The composition of this kind of effluent is influenced by various factors such as the amount, composition and moisture of the solid waste, age of the landfill, hydrogeology and climate of the site and seasonal weather variations [9]. This variability requires a flexible and broad-ranging treatment system, which results in increased costs. Some common aspects that can be identified among leachates of different ages and sites are the high levels of N–NH_4_^+^, the high chemical oxygen demands and the low phosphate concentrations [10,11].

As was reported by several authors, leachate treatment can be performed using conventional wastewater treatment processes or membrane processes [1,10]. The simplest and cheapest conventional options are recycling [12], combined treatment with domestic wastewater [13,14], and lagooning with recycling. More complex conventional techniques include the biological (aerobic, anaerobic, or mixed) and the chemical/physical processes, such as coagulation-flocculation, chemical precipitation and air stripping. Among the membrane processes, physical porous barriers can be used to retain certain sized particles from the leachate (microfiltration, ultrafiltration, or nanofiltration). The use of semipermeable membranes coupled with the induction of differences in solute concentrations and pressure levels can also be used with the goal of decreasing concentrations of certain ions, molecules and/or particles (reverse osmosis). The possibility of using just one of the cited processes to produce a safe and legally dischargeable treated effluent is remote. Accordingly, different techniques must be combined in order to effectively treat a landfill leachate. Classical treatments are currently insufficient in achieving high depuration levels, mainly due to the increasing discharge restrictions and standards [1]. More recently, with the advances on membrane processes, higher treatment effectiveness has been achieved, but with substantially greater costs and subsequent problems (i.e., the production of an unusable concentrate and membrane fouling). In response to these flaws, sustainable remediation technologies that allow for the reduction of the treatment operational costs must be developed, optimized and implemented.

Phycoremediation is a suitable option, since it combines metabolic incorporation of pollutants with biomass production, which in turn can be used as feedstock for biofuels production [15]. By definition, phycoremediation is the process of depuration of waste or wastewater carried out by micro- and macroalgae [16,17], not only in terms of nitrogen and phosphorus (which are macronutrients), but also in terms of heavy metals and organic pollutants. It can be used as a complementary step to existing treatment systems that produce effluents that still have high concentrations of inorganic nitrogen and phosphorus (tertiary treatment). The use of this kind of bioremediation process in leachate treatment is not yet well developed. Its utilization is still dependent on a set of conditions and cannot be done independently. For example, Cheung, et al. [18] proved that microalgal growth in leachates without inhibition is only possible by avoiding the acute toxicity effects of the inhibitory compounds present in the leachate, which can be accomplished through a dilution in water or wastewater or by using specific pollutant-tolerant species. Even though some of these compounds are essential micronutrients in trace concentrations (e.g., metals) and can be obtained from the culture medium, they can have severe toxic effects by metabolic interference if present at high concentrations [19,20]. This sustainable depuration process has proved to be beneficial and efficient by several authors in a variety of test conditions [21,22,23,24,25,26,27] and it is now a growing area of interest in biological wastewater treatment. The fact that precursors of biofuels can be produced with these processes also makes them appealing to the biofuels industries, since they can be used as a sustainable low-cost input source of raw-materials.

The growth of microalgae in leachates must be studied under a variety of conditions to allow a standardization of approaches in the future. Therefore, the main goal of the present study was to evaluate biomass production and nutrient uptake by *C. vulgaris* cultivated in different compositions of a pre-treated leachate, with special focus on nitrogen consumption. Additionally, the feasibility of using this biological process as a complementary treatment step for an existing system was also assessed.

## 2. Results

### 2.1. Biomass Production

**Figure 1:**
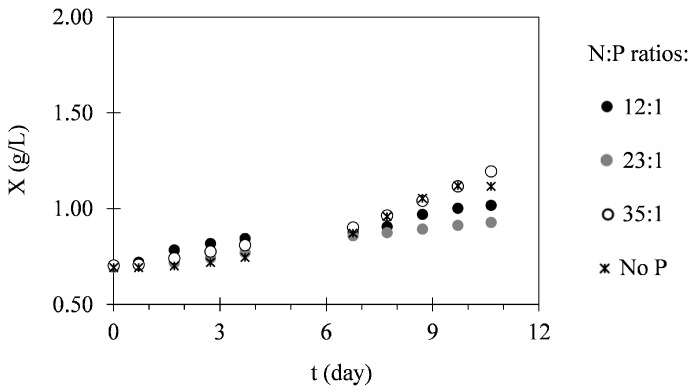
Temporal variation of biomass concentrations determined for assay III under different N:P ratios. The presented data correspond to the mean obtained from two independent experiments.

Maximum biomass concentrations, average biomass productivities and specific growth rates determined for the assays I, II, and III are shown in Table 1. Analysis of these data suggests that *C. vulgaris* growth was favoured in cultures performed in leachates presenting lower N–NH_4_^+^ concentrations. Figure 1 presents an example of the growth curves obtained in this study for assay III (the one presenting the highest nitrogen concentration). According to these data, *C. vulgaris* growth curves in the different N:P ratios and with no phosphorus addition show similar behaviour.

### 2.2. Nutrient Uptake

**Figure 2:**
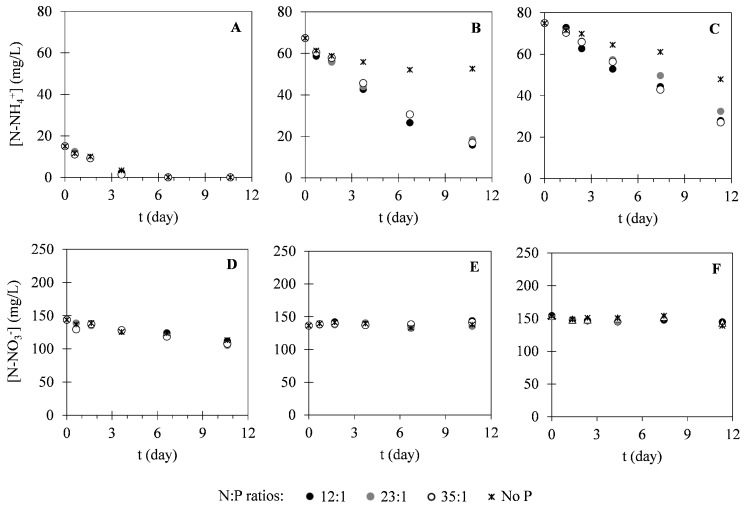
Temporal variation of N–NH_4_^+^ (**A**–**C**) and N–NO_3_^−^ (**D**–**F**) concentrations determined in assays I (**A** and **D**), II (**B** and **E**) and III (**C** and **F**) under different N:P ratios. The presented data correspond to the mean obtained from two independent experiments.

**Figure 3:**
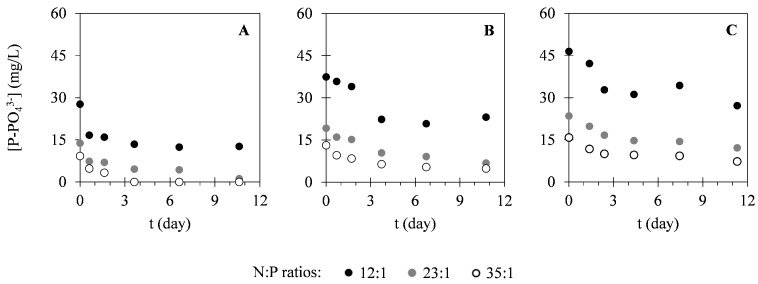
Temporal variation of P–PO_4_^3−^ concentrations determined in assays I (**A**), II (**B**) and III (**C**) under different N:P ratios. The presented data correspond to the mean obtained from two independent experiments.

**Figure 4:**
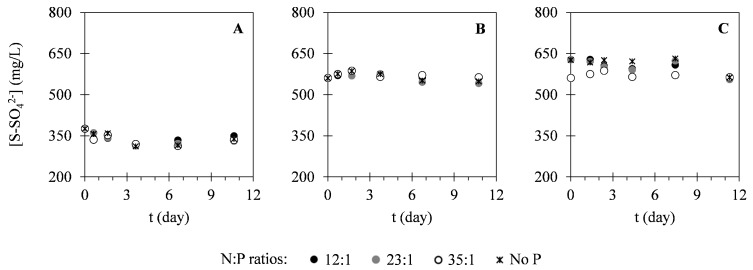
Temporal variation of S–SO_4_^2−^ concentrations determined in assays I (**A**), II (**B**) and III (**C**) under different N:P ratios. The presented data correspond to the mean obtained from the two independent experiments.

**Figure 5:**
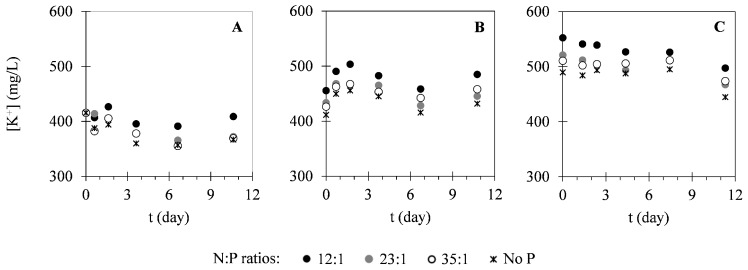
Temporal variation of K^+^ ion concentration in assays I (**A**), II (**B**) and III (**C**) under different N:P ratios. The presented data correspond to the mean obtained from the two independent experiments.

**Figure 6:**
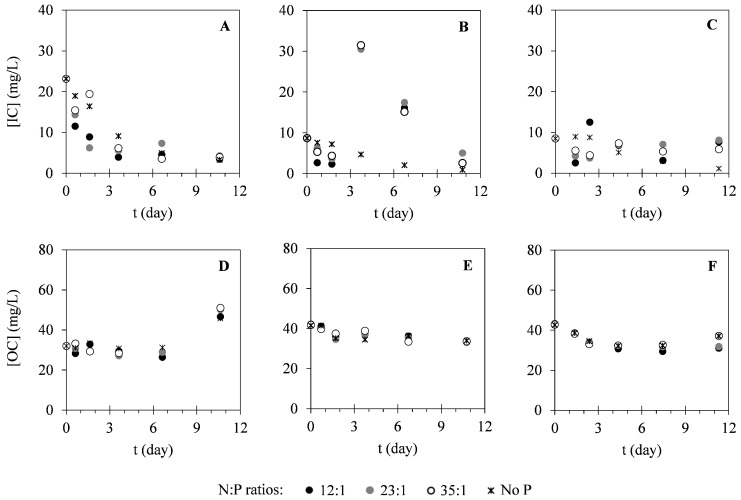
Temporal variation of IC (**A**–**C**) and OC (**D**–**F**) concentrations determined in assays I (**A** and **D**), II (**B** and **E**) and III (**C** and **F**) under different N:P ratios. The presented data correspond to the mean obtained from the two independent experiments.

In this study, different nutrients were monitored within the cultivation time to evaluate the potential of *C. vulgaris* in nutrients uptake from different compositions of landfill leachate. Figure 2 presents the variation of nitrogen concentration in the forms of N–NH_4_^+^ and N–NO_3_^−^. As it is possible to see in Figure 2, N–NH_4_^+^ concentrations decreased within the cultivation time. On the other hand, N–NO_3_^−^ concentrations remained approximately constant during the cultivation period. Regarding phosphorus (P–PO_4_^3−^) concentration, variation of this parameter can be observed in Figure 3. These results show that P–PO_4_^3−^ concentrations also decreased within the cultivation time. Figure 4 presents the variation of sulphur (S–SO_4_^2−^) concentration in microalgal cultures, evidencing only a slight decrease during the cultivation period. Similarly, the potassium ion (K^+^) concentration has also shown a slight decrease, as it is represented in Figure 5. Inorganic and organic carbon (IC and OC, respectively) concentrations were also monitored with the purpose of verifying the metabolism adopted by *C. vulgaris*, which has shown to be mainly autotrophic (Figure 6).

The experimental data regarding nutrients concentrations in the culture medium from each culture were used for the determination of removal efficiencies and average removal rates. Additionally, in the case of nitrogen (N–NH_4_^+^) and phosphorus removal, pseudo-first-order kinetic constants and biomass yields based on these nutrients consumption were determined. These parameters are presented in Table 2 (in the case of nitrogen and phosphorus) and Table 3 (in the case of sulphur and potassium ion).

## 3. Discussion

### 3.1. Biomass Production

The daily monitoring of biomass concentration allowed the evaluation of *C. vulgaris* growth under different conditions. Figure 1 shows the different growth phases of *C. vulgaris* during 12 days of culture. The adaptation phase was generally short, similar to those already reported in the literature for this microalga [28,29]. This can be explained by the fact that these cultures were previously adapted to grow in a landfill leachate [30]. In the exponential phase, microalgal growth suggests some sort of growth limitation, either from a shading phenomenon or from difficulties in nutrient transfer from the medium to the cell interior [31]. Another possible inhibition factor could be the presence of heavy metals in the effluent. However, according to the literature [32,33,34], the concentrations of these pollutants were not high enough to negatively affect microalgal growth. In assay II, the presence of phosphorus has proved to be an enhancement aspect, since the supplemented cultures presented increased growth in the same period of time. This can be observed in Table 1, where it is possible to determine an increase in average biomass productivities of supplemented cultures of about 86% when compared to that of non-supplemented cultures.

With lower nutrient concentrations and similar initial biomass concentrations, the cultures from assay II performed better than those of assay III, indicating that high concentrations of some of the nutrients present in the leachate can negatively impact *C. vulgaris* growth. These results reinforce the hypothesis of Cheung, et al. [18], which states that microalgal growth in leachate is only possible by avoiding the acute toxicity of the inhibitory compounds, for example, ammonium when present at high concentrations [35].

The determined average biomass productivities (0.020–0.11 g·L^−1^·day^−1^) were similar to those observed by Griffiths, et al. [36] when growing *C. vulgaris* on a synthetic medium (0.016–0.373 g·L^−1^·day^−1^). In the specific case of assays I and II, average biomass productivities are situated amongst the array of values reported by Silva, et al. [37] when using a synthetic wastewater as growth medium. On the other hand, specific growth rates (0.028–0.13 day^−1^) were globally low when compared to typical values reported in the literature of 0.11 and 1.37 day^−1^ [38,39]. The lower specific growth rates observed in assays II and III can be explained by a self-shading phenomenon, since significantly higher inoculation concentrations were used in these assays.

### 3.2. Nutrient Uptake

For nitrogen (N–NH_4_^+^ and N–NO_3_^−^), it was expected that N–NH_4_^+^ would be the preferential inorganic species to be consumed by *C. vulgaris*, since it is the only species whose assimilation does not involve oxidation-reduction reactions. This trend has already been reported by several authors [37,40,41] and it was also demonstrated in this study, as can be seen in Figure 2 (no adaptation phase was observed). Total N–NH_4_^+^ removal was achieved in assay I, pairing with considerable N–NO_3_^−^ removal efficiencies of about 21%–27%. On the other hand, minimum variations in N–NO_3_^−^ concentrations were observed in the other assays (no more than 10%), along with appreciable N–NH_4_^+^ removal efficiencies (up to 77%). The differences observed for the different N:P ratios indicate that nitrogen removal was enhanced by the addition of phosphorus to the cultures. Silva, et al. [37] have reported N–NH_4_^+^ kinetic constants ranging between 0.19 and 3.86 day^−1^, which are higher than those determined in the assays II and III. These results are associated with the lower specific growth rates in these assays caused by the self-shading phenomenon. On the other hand, kinetic constants determined for the assay with the lowest initial N–NH_4_^+^ concentration (assay I) were close to those reported in the referred study. Similarly, the removal rates of total nitrogen determined in this study (1.2–5.1 mg·L^−1^·day^−1^) have already been reported for *C. vulgaris* in the literature [38,39,42,43,44]. Silva, et al. [37] have also determined biomass yields based on nitrogen consumption, obtaining values between 13.5 and 75.2 g_X_·g_N_^−1^. These values are similar to those determined in this study for assays I and II. However, lower values were obtained for assay III, which is in accordance with the kinetic growth parameters determined in the experiments concerning this leachate composition. Regarding phosphorus consumption, removal rates obtained (0.76–1.7 mg·L^−1^·day^−1^) were higher than the range of rates already reported in the literature (0.07–0.52 mg·L^−1^·day^−1^) [38,44]. This can be explained by other phosphorus removal mechanisms rather than assimilation. In microalgal cultures, phosphorus removal can also occur through chemical precipitation (at pH values higher than 8) or through luxury uptake, a mechanism adopted by microalgae that consists of the assimilation of high phosphorus amounts and storage in the form of polyphosphates [37,45,46]. In the studied cultures, pH values were close to 8 (data not shown), meaning that some phosphorus precipitation might have occurred. The low biomass yields based on phosphorus consumption obtained in this study (20–150 g_X_·g_P_^−1^) also indicate that other phosphorus removal mechanisms might have occurred. Regarding the kinetic constants, values obtained in this study for the N:P ratios of 12:1 and 23:1 of assay I (0.16 and 0.20 day^−1^, respectively) were similar to those reported by Wang, et al. [47] (0.17–0.32 day^−1^). On the other hand, a kinetic constant of 0.6 day^−1^ was obtained for the N:P ratio of 35:1. In the assays II and III, low values were obtained for the kinetic constants (0.043–0.11 day^−1^), indicating that the pseudo-first-order kinetic model might be inadequate to describe phosphorus uptake in these conditions.

Even though reasonable removal efficiencies of nitrogen and phosphorus were achieved, according to the European Union (EU) legislation for wastewater deposition [48,49], the final concentrations in all assays were insufficient to allow a safe and legal discharge of the treated leachate (emission limits established by EU legislation are 15 mg_N_·L^−1^ and 2 mg_P_·L^−1^ for nitrogen and phosphorus, respectively) [48,49]. Despite the fact that the addition of phosphorus potentiates biomass growth and nitrogen removal, it is indeed a delicate step. That is, if a scale-up of a phycoremediation process is to be done with the addition of phosphorus, the treatment time must be sufficient to allow a reduction of the added concentration until at least the legal emission limit is achieved. Otherwise, the addition step will end up polluting the wastewater that is being treated. Further developments must be done in this regard in order to assess the conditions in which the process can be viable.

With respect to sulphur, its initial concentration in all cultures was already below the EU emission limit (668 mg_S_·L^−1^) [48,49]. Accordingly, sulphur removal was not as imperative as nitrogen and phosphorus removal in the designed assays. Although it is an important element for microalgae, being present in essential amino acids [50], its assimilation is still a poorly documented topic and the currently available information is limited to very few species [51]. Analysing the variations of sulphate concentrations (Figure 5) and the calculated removal efficiencies (Table 3), it is possible to conclude that no significant removal occurred in any case, since the variations were almost negligible (maximum removal efficiencies obtained were 12%). However, removal rates determined in the present study (1.1–11 mg·L^−1^·day^−1^) were significantly higher than those already reported in the literature (0.275–0.543 mg·L^−1^·day^−1^) [37]. This discrepancy can be explained by the differences observed between the initial conditions used in this study and those of the cited one. Comparing the removal efficiencies and removal rates obtained for the different N:P ratios evaluated, it is possible to conclude that the addition of phosphorus did not contribute to increased sulphur removal.

Lastly, potassium ion removal was also insignificant. A maximum removal efficiency of 12% and a maximum removal rate of 4.9 mg·L^−1^·day^−1^ were achieved in assays I and III, respectively. Even though it plays a crucial role in osmotic regulation of microalgal cells and protein synthesis processes [52], little is known about its assimilation by microalgae.

Regarding the variation of IC and OC concentrations present in the culture medium, the decrease observed in the IC concentration indicates the assimilation of this carbon source during photosynthetic growth of the studied microalga. However, in assay III, where initial IC concentration was lower than in the other assays, a decrease in OC concentration was also observed. This might be due to the mixotrophic growth of *C. vulgaris*. Although microalgal growth is mainly autotrophic, when both IC and OC are present in the culture medium, microalgae perform both photosynthesis and oxidative assimilation [53,54].

## 4. Materials and Methods

### 4.1. Landfill Leachates

Landfill leachates evaluated in this study (corresponding to assays I, II and III) resulted from different batches of a sanitary landfill located in the north of Portugal. They were collected at the exit of an aerated stabilization pond and subjected to three of the four stages of a patented treatment process [55]: (i) biological oxidation in anoxic and aerobic regimes; (ii) coagulation-flocculation stage with iron(III) chloride (240 mg_Fe(III)_·L^−1^) at pH 4.2, followed by 12-h sedimentation; and (iii) a photo-oxidation process using natural sunlight (2.08 m^2^ of Compound Parabolic Collectors), by a photo-Fenton reaction, with the addition of iron(II) sulphate and hydrogen peroxide, followed by a neutralisation step.

Since the leachate did not contain significant amounts of inorganic phosphorus, additions of KH_2_PO_4_ were made in order to evaluate microalgal growth under different N:P molar ratios of 12:1, 23:1 and 35:1. Parallel experiments without phosphorus addition were also conducted. The empiric Redfield ratio (16:1) was in the base of the ratio selection, since it describes the atomic composition usually found in aquatic photosynthetic microorganisms [56]. Table 4 presents the chemical composition of leachates I, II and III. These effluents also contained the following heavy metals: cadmium (up to 0.005 mg·L^−1^), copper (up to 0.005 mg·L^−1^), lead (up to 0.005 mg·L^−1^), chromium (up to 1.1 mg·L^−1^) and zinc (up to 0.3 mg·L^−1^). Research studies showed that these concentrations do not significantly affect microalgal growth [32,33,34]. In addition, microalgae were able to assimilate these nutrients and to remove them from wastewater.

### 4.2. Microalgal Cultivation

The used species was *C. vulgaris* since the growth and nutrient-consumption rates associated with its cultivation on different wastewaters are favourable, as demonstrated by several authors [37,38,39,42,43,44]. The used strain (*C. vulgaris* CCAP 211/11B) was obtained from the United Kingdom’s Culture Collection of Algae and Protozoa. Cultivation was performed in 1-L borosilicate glass flasks under continuous exposure to a photon flux density of 32–42 μmol·m^−2^·s^−1^ obtained by a set of fluorescent lightbulbs. Mixing was guaranteed by atmospheric air injection at approximately 90 L·h^−1^ using TARP D-2463 air pumps (Trixie, Tarp, Germany). The injected air along with the superficial area (75 cm^2^) were the only options for gas-transfer processes between the cultures and the atmosphere. To avoid carbon dioxide stripping at pH levels lower than 7 [57], Na_2_CO_3_ was added to the cultures whenever their pH levels were low. To evaluate the effect of different leachate compositions on microalgal growth and nutrient uptake, the leachates I, II and III were used as culture medium, thus resulting in assays I, II and III. Additionally, for each of these assays, different concentrations of phosphorus were added, so that N:P molar ratios were approximately 12:1, 23:1 and 35:1. Microalgal growth with no phosphorus addition was equally evaluated. For each condition, two independent experiments were performed at room temperature, being the average of the culture temperatures 16 ± 2, 20 ± 2 and 21 ± 2 °C for the assays I, II and III, respectively. *C. vulgaris* previously grown in the leachate and presenting the lowest nitrogen concentration (leachate I) with a N:P ratio ranging between 20:1 and 24:1 was used as inoculum.

### 4.3. Analytical Methods

Culture pH and temperature were monitored daily using a HI 8424 sensor (HANNA Instruments, Vöhringen, Germany). On the other hand, illuminance measurements were performed during day-time and night-time periods using a ISO-TECH LUX-1335 device (RS Components, Corby, UK). Optical density (OD) at 440 nm was also measured on a daily basis using a Spectroquant Pharo 100 spectrophotometer (Merck, Lisbon, Portugal). Selection of this wavelength was based on the fact that maximum absorbance for the genus *Chlorella* at this value has already been reported [58]. At the same time, 40-mL samples were collected during the cultivation period to determine biomass concentrations (X) in terms of cell dry weight. OD measurements, together with cell dry weight values were used to determine a calibration curve between these variables (data not shown), so that growth monitoring could be easily assessed through the OD measurements. Coefficients of determination obtained for these models were higher than 0.992.

On days 0, 1, 2, 4, 7 and 11, 10-mL samples from each of the cultures were collected, centrifuged at 4000 rpm for 15 min using a Himac CT66 centrifuge (VWR, Carnaxide, Portugal) and filtered using 0.45-μm nylon membranes. Inorganic anions (NO_3_^−^, NO_2_^−^, PO_4_^3−^ and SO_4_^2−^) were determined by ion chromatography by injecting 10 µL of sample into a Dionex ICS-2100 LC (Thermo Scientific Dionex, Linda-a-Velha, Portugal) equipped with an IonPac^®^ AS11-HC 250 mm × 4 mm column (Thermo Scientific Dionex) at 30 °C and an anion self-regenerating suppressor ASRS^®^ 300, 4 mm (Thermo Scientific Dionex) under isocratic elution of 30 mM NaOH at a flow rate of 1.5 mL·min^−1^. Inorganic cations (NH_4_^+^ and K^+^) were also determined by ion chromatography by injecting 25 µL of sample into a Dionex DX-120 LC (Thermo Scientific Dionex) equipped with an IonPac^®^ CS12A 250 mm × 4 mm column (Thermo Scientific Dionex) at ambient temperature and a cation self-regenerating CSRS^®^ Ultra II, 4 mm (Thermo Scientific Dionex) suppressor under isocratic elution of 20 mM methanesulfonic acid at a flow rate of 1.0 mL·min^−1^. Total dissolved carbon (TC) and dissolved inorganic carbon (IC) were measured in a TOC-V_CSN_ analyser (Shimadzu, Duisburg, Germany) equipped with an ASI-V autosampler and dissolved organic carbon was determined by the difference between TC and IC.

### 4.4. Kinetic Models and Parameters

Biomass concentration values allowed the determination of specific growth rates (*μ*, day^−1^) and average biomass productivities (*P_X_*, g·L^−1^·day^−1^) according to Equations (1) and (2), respectively.

(1) $$\frac{dX}{dt}=\mu \times X\Leftrightarrow \mu =\frac{ln\left(X_{1}/X_{0}\right)}{t_{1}-t_{0}}$$

where *t*_1_ and *t*_0_ represent the end and the beginning, respectively, of the exponential growth phase and *X*_1_ and *X*_0_ correspond to the biomass concentrations at time-steps *t*_1_ and *t*_0_, respectively.

(2) $$P_{X}=\frac{X_{f}-X_{i}}{t_{f}-t_{i}}$$

where steps *t_f_* and *t_i_*, represent the end and the beginning, respectively, of the cultivation period, and *X_f_* and *X_i_* correspond to the biomass concentrations at time-steps *t_f_* and *t_i_*, respectively.

Regarding nutrient uptake by microalgae, removal efficiencies (*RE*, %) and average removal rates (*RR*, mg·L^−1^·day^−1^) of all the considered nutrients were calculated using Equations (3) and (4).

(3) $$RE=\frac{S_{0}-S_{f}}{S_{0}}$$

(4) $$RR=\frac{S_{0}-S_{f}}{t_{f}}$$

where *S*_0_ and *S_f_* correspond to the initial and final substrate concentrations, respectively, and *t_f_* corresponds to the total time of the experiment.

The temporal evolutions of ammonium and phosphate concentrations were assumed to follow a pseudo-first-order kinetic model, represented by Equation (5), in which *S*_0_ corresponds to the substrate initial concentration. The linearization of this equation allowed the determination of kinetic constants (*k*, day^−1^).

(5) $$\frac{dS}{dt}=k\times t\Leftrightarrow ln\left(S\right)=ln\left(S_{0}\right)-\;k\times t$$

Using the different values of average biomass productivities and removal rates, specific biomass yields based on nutrients consumption (*Y_X/S_*, g_X_·g_S_^−1^) were determined (Equation (6)). These values represent the biomass produced for the mass of substrate consumed.

(6) $$Y_{X/S}=\frac{P_{X}}{RR}$$

## 5. Conclusions

In this study, *C. vulgaris* growth in different compositions of a landfill leachate was evaluated. At the same time, phosphorus was added to the landfill leachate at different concentrations so that different N:P ratios could be evaluated (12:1, 23:1 and 35:1). Together with microalgal growth, the nutrient uptake ability of the microalgae was also assessed. For this, concentrations of different nutrients (nitrogen, phosphorus, sulphur and potassium ion) were monitored within the cultivation period to allow the characterization of nutrient uptake kinetics. The results have shown that *C. vulgaris* was able to grow in different formulations of the landfill leachate. However, increased growth was observed in the experiments performed with the lowest N–NH_4_^+^ concentration (assay I). Additionally, this study has shown that microalgal growth was favoured by the addition of phosphorus to the culture medium. Landfill leachate typically presents a high N:P ratio, which can be harmful for microalgal growth and thus phosphorus supplementation prior to microalgal culturing should be added to promote an effective removal of the excessive nitrogen. Regarding nutrient uptake, an effective removal of N–NH_4_^+^ was observed in all the experiments, especially in those supplied with phosphorus. On the other hand, only a slight decrease in N–NO_3_^−^ concentrations was observed, which may be related to the higher affinity of microalgae to N–NH_4_^+^. Phosphorus removal from the culture medium was also observed. However, the low yields on biomass based on phosphorus consumption suggest another removal mechanism rather than P–PO_4_^3−^ assimilation. Taking into account the obtained results, it is possible to conclude that *C. vulgaris* growth in landfill leachate for remediation purposes can be effectively used. However, it should be noted that it must not contain high levels of toxic compounds, such as high N–NH_4_^+^ concentrations and there must be enough phosphorus present to avoid growth limitation due to low phosphorus levels.

## Figures and Tables

**Table 1:** Biomass production parameters determined for assays I, II and III under different N:P ratios.

Assay	N:P Ratio	*X_i_* (g·L^−1^)	*X*_max_ (g·L^−1^)	*P_X_* (g·L^−1^·Day^−1^)	*μ* (Day^−1^)
I	12:1	0.194	0.91 ± 0.03 ^a^	0.107 ± 0.004	– ^b^
	23:1	0.194	0.83 ± 0.03 ^a^	0.095 ± 0.004	– ^b^
	35:1	0.194	0.86 ± 0.03 ^a^	0.101 ± 0.005	– ^b^
	No P	0.194	0.81 ± 0.09 ^a^	0.09 ± 0.02	– ^b^
II	12:1	0.607	1.52 ± 0.05	0.0988 ± 0.0004	0.13 ± 0.02
	23:1	0.606	1.71 ± 0.06	0.11 ± 0.09	0.099 ± 0.005
	35:1	0.607	1.70 ± 0.05	0.11 ± 0.02	0.109 ± 0.003
	No P	0.608	1.44 ± 0.08	0.057 ± 0.002	0.068 ± 0.002
III	12:1	0.701	0.970 ± 0.004	0.034 ± 0.003	0.060 ± 0.007
	23:1	0.695	0.894 ± 0.007	0.020 ± 0.006	0.028 ± 0.003
	35:1	0.704	1.04 ± 0.02	0.049 ± 0.009	0.0724 ± 0.0007
	No P	0.693	1.06 ± 0.01	0.038 ± 0.002	0.085 ± 0.007

*X_i_*—initial biomass concentration (g·L^−1^); *X*_max_—maximum biomass concentration (g·L^−1^); *P_X_*—average biomass productivity (g·L^−1^·day^−1^); *μ*—specific growth rate (day^−1^). ^a^ Maximum biomass concentrations determined for assay I correspond to those obtained on the seventh day of culturing; ^b^ Specific growth rates for the assay I were not determined because it was not possible to obtain enough data corresponding to the exponential growth phase.

**Table 2:** NH_4_^+^, NO_3_^−^ and PO_4_^3−^ removal parameters determined for assays I, II and III under different N:P ratios.

Assay	N:P Ratio	N–NH_4_^+^ *k* (Day^−1^)	N–NH_4_^+^ *RE* (%)	N–NO_3_^−^ *RE* (%)	Total-N *RR* (mg·L^−1^·Day^−1^)	*Y_X/N_* (g_X_·g_N_^−1^)	P–PO_4_^3−^ *k* (Day^−1^)	P–PO_4_^3−^ *RE* (%)	P–PO_4_^3−^ *RR* (mg·L^−1^·Day^−1^)	*Y_X/P_* (g_X_·g_P_^−1^)
I	12:1	0.51 ± 0.08	100%	22%	4.4	24 ^a^	0.16 ± 0.08	54%	1.4	76 ^a^
	23:1	0.7 ± 0.2	100%	27%	5.1	19 ^a^	0.20 ± 0.03	92%	1.2	80 ^a^
	35:1	0.7 ± 0.2	100%	25%	4.8	21 ^a^	0.6 ± 0.2	100%	0.87	116 ^a^
	No P	0.41 ± 0.06	100%	21%	4.3	22 ^a^	–	–	–	– ^a^
II	12:1	0.135 ± 0.005	77%	<0%	4.1	24	0.089 ± 0.006	38%	1.3	74
	23:1	0.120 ± 0.003	73%	1%	4.7	23	0.093 ± 0.007	65%	1.2	95
	35:1	0.128 ± 0.006	75%	<0%	4.2	27	0.09 ± 0.02	63%	0.77	150
	No P	0.034 ± 0.007	22%	<0%	1.2	46	–	–	–	–
III	12:1	0.091 ± 0.007	63%	6%	5.0	6.8	0.045 ± 0.005	41%	1.7	20
	23:1	0.080 ± 0.002	57%	9%	5.0	4.1	0.11 ± 0.02	48%	1.0	20
	35:1	0.090 ± 0.006	64%	7%	5.1	9.5	0.043 ± 0.008	54%	0.76	64
	No P	0.040 ± 0.002	36%	10%	3.7	10	–	–	–	–

*k*—kinetic constant (day^−1^); *RE*—removal efficiency (%); *RR*—average removal rate (mg·L^−1^·day^−1^); *Y_X/N_*—biomass yield on nitrogen consumption (g_X_·g_N_^−1^); *Y_X/P_*—biomass yield on phosphorus consumption (g_X_·g_N_^−1^). ^a^ Specific yields of assay I were obtained using average biomass productivities determined for the first seven days of culturing.

**Table 3:** SO_4_^2−^ and K^+^ ion removal parameters determined for assays I, II and III under different N:P ratios.

Assay	N:P Ratio	S–SO_4_^2−^ *RE* (%)	S–SO_4_^2−^ *RR* (mg·L^−1^·Day^−1^)	K^+^ *RE* (%)	K^+^ *RR* (mg·L^−1^·Day^−1^)
I	12:1	7%	2.5	2%	0.63
	23:1	12%	4.3	10%	4.1
	35:1	11%	3.9	11%	4.3
	No P	10%	3.5	12%	4.6
II	12:1	0%	<0	<0%	<0
	23:1	4%	1.9	<0%	<0
	35:1	0%	<0	<0%	<0
	No P	2%	1.1	<0%	<0
III	12:1	11%	6.0	10%	4.9
	23:1	11%	6.4	10%	4.7
	35:1	8%	4.4	7%	3.3
	No P	10%	5.6	9%	4.0

*RE*—removal efficiency (%); *RR*—average removal rate (mg·L^−1^·day^−1^).

**Table 4:** Chemical composition of the different landfill leachates used in the assays I, II and III.

Assay	[N–NH_4_^+^] (mg·L^−1^)	[N–NO_3_^−^] (mg·L^−1^)	[P–PO_4_^3−^] (mg·L^−1^)	[S–SO_4_^2−^] (mg·L^−1^)	[K^+^] (mg·L^−1^)
I	15	144	<0.1	377	416
II	67	136	1	561	412
III	75	153	1	627	490
